# Cocreating a Mobile Health App Providing Physical Activity Recommendations for Older People Living With Parkinson Disease or Dementia: User-Centered Pilot Study

**DOI:** 10.2196/51831

**Published:** 2025-06-19

**Authors:** Ellen Bentlage, Alberto del Río Ponce, Mona Ahmed, Pilar Gangas, Michael Brach, Jorge Alfonso Kurano, José Manuel Menéndez

**Affiliations:** 1 Insitute of Sport and Exercise Sciences, University of Münster, Horstmarer Landweg 50, Münster, 48149, Germany, 49 251 83-34889; 2 Grupo de Aplicación de Telecomunicaciones Visuales, Universidad Politécnica de Madrid, Madrid, Spain; 3 Annexe Offices, International Foundation for Integrated Care, Oxford, United Kingdom; 4 CSIC-UAC Técnico de Asesoramiento Cientifico, CSIC-UAC, Madrid, Spain

**Keywords:** healthcare, physical activity, personalized recommendations, ICT, quality of life, codesign process

## Abstract

**Background:**

The project “Personalized Integrated Care Promoting Quality of Life for Older People” aimed to develop an integrated care system based on information and communication technology to support older people living with Parkinson disease or dementia disease. One module focuses on physical activity (PA) recommendations.

**Objective:**

The objective of the study is to describe the development process of the PA recommendation system from the behavior-change and technical perspective, followed by its content and satisfaction evaluation.

**Methods:**

This study describes the development of the PA recommendations based on the Health Action Process Approach (HAPA). A first pilot assessed the feasibility of the overall PROCare4Life system (previously reported). In a second pilot, users evaluated the content of the PA recommendations during 40 intervention days. In a third pilot, users evaluated their satisfaction with a mobile health satisfaction questionnaire.

**Results:**

The PA recommendations focused on different aspects of an adapted version of the HAPA model, while they simultaneously approached 3 activation factors: skills, knowledge, and motivation. The content was generally well-received, with most users rating key sections as excellent or good, particularly “benefits and consequences of PA” (34/43, 79%) and “five golden rules of PA” (34/41, 83%). However, less than a third gave high ratings to “PA guidelines of the WHO” (9/36, 25%) and “practical tips for PA” (10/35, 29%). Regarding satisfaction, at least half of the 237 participants found it easy and good to use, with acceptable time spent and clear instructions. Compared with agreement or neutral evaluations, most disagreed with negative statements about it being time-consuming (111/237, 47%) or boring (99/237, 42%). While 41% (97/237) recommended it and 44% (104/237) felt it helped them understand lifestyle benefits, fewer agreed the recommender system helped them set personal goals (78/237, 33%) or motivated change (88/237, 37%). Users found the recommendations understandable, engaging, and practical, though some aspects, such as motivation and goal setting, received criticism. Challenges in pilot 2, particularly related to setup difficulties and limited participation, led to system modifications in pilot 3 that improved usability and data collection.

**Conclusions:**

It has been confirmed that cocreating and iteratively testing the contents on HAPA and approaching the activation factors contributed to increasing the acceptance of the PA. The intervention development was based on user needs and used comparable methodology across user profiles and pilot phases. All in all, users were positive about the content. The research team has identified that digital systems, that provide monitoring functions of the mobile health app and Fitbit wristband are considered advantageous by participants in the cocreation process. Addressing the activation factors can be recommended for researchers and technical developers of other projects. Future adjustments to the design should focus on personalization to encourage the adoption of a healthier lifestyle.

## Introduction

### Active and Inactive Lifestyles

The World Health Organization has developed guidelines for different age groups on how much physical activity (PA) is needed for good health [1], whereby adults should get 150 minutes of moderate PA or 75 minutes of vigorous PA per week. Yet, on a validated PA questionnaire answered by over 2000 older adults in the European Union (mean age of 74.9, SD 4.5 years), 42% were found to not meet these global PA recommendations. These findings mirror others indicating that most older adults have a physically inactive and sedentary lifestyle [2-4]. Further, older participants tend to meet the recommendations less often [5]. Notably, people’s amount of PA decreased dramatically during the COVID-19 pandemic, when they faced isolation and social distancing. Specifically, home confinement reduced people’s total weekly PA time and intensity and increased daily sitting time in a health-threatening direction [6], as these factors increase one’s risk of mortality, obesity, sarcopenia, frailty, falls, functional impairments, and neurodegenerative diseases [7,8]. By contrast, regular PA results in a lower risk of falls and mortality as well as an improved overall health status, including better Quality of Life (QoL) [7,9]. PA is known to reduce one’s risk of dementia disease (DD) [10], and in Parkinson disease (PD) it is “a vital component to maintain balance, mobility and activities of daily living” [11]. In a web-based survey from May to August 2020, 75% (64/85) of participants with these conditions reported that receiving PA recommendations was desired, and 79% (57/72) of caregivers reported the same. Interviews with patients and workshops with health care professionals support these results [12].

### Behavior Change Techniques

Strategies for changing people’s behavior to become healthier fall under the general umbrella of behavior change techniques, which might often rely on 3 activation factors: skills, knowledge, and motivation. Research shows that previous interventions that addressed these factors could positively influence individuals’ PA behavior [13,14]. Notably, these factors can be addressed by different behavior change techniques; for example, skills can be trained with instructions or demonstrations of a specific exercise [15], and knowledge can be addressed by educating individuals on the benefits of sufficient PA and the consequences of insufficient PA and by comparing their own PA behavior with guidelines [16], and motivation can be targeted by activity monitoring, either by the user, another person or a technical device [16], and through personalized feedback [17]. All of these were incorporated into PROCare4Life mobile app and iteratively tested and cocreated with older people living with PD or dementia. A recent review showed that integrating the abovementioned activation factors results in the most benefits when aiming at influencing PA behavior. Further, addressing the 3 activation factors can be accomplished by using a variety of interfaces [18] and digital interactions, that have been incorporated into PROCare4Life mobile app. Thus, it can be expected according to previous literature that digital health systems incorporating PA recommendations can potentially benefit from incorporating the above-mentioned activation factors and using various modalities to do so. These expectations were tested through the cocreation process of the PROCare4Life mobile app, which includes PA recommendations. The PROCare4Life PA recommendations approached skills and knowledge via notifications and PDFs that were displayed on the users’ smartphones, whereas the motivation was approached by personalized gamification of the smartwatch app, which could also be read through the mobile app. In addition, the PA recommendations were developed according to the Health Action Process Approach (HAPA). HAPA was used to guide the development of PROCare4Life PA training and adherence programs from a psychological theory perspective to enhance the efficiency of PA behavioral changes among older people living with PD or dementia (Alzheimer disease and others) [19].

### PROCare4Life and Health Recommender Systems

In the European Union, the recent project “Personalized Integrated Care Promoting Quality of Life for Older People” (PROCare4Life) funded by Horizon 2020 (grant agreement number 875221, 2020‐2023) developed an interactive care system based on digital technologies for people living with neurodegenerative diseases, their caregivers (mobile health app, smartwatch for both profiles), and health care professionals (web interface) [20]. Health recommender systems [19] have become valuable digital tools [21-23], as they provide personalized suggestions and predictions to users by analyzing vast amounts of data and using advanced algorithms [24]. Health recommender systems have shown significant potential to improve patients’ and caregivers’ healthy behaviors and QoL [25-27]. The cocreation and iterative testing supported the development of the digital interactive care system through several pilot waves. PROCare4Life pilots were implemented at 6 real-life sites including daycare, home, and rehabilitation scenarios, located in 5 different EU (European Union) countries (Germany, Italy, Portugal, Romania, and Spain). The cocreation methodology included several steps:

1. A user requirements study [12,28] in which PROCare4Life users shared their needs linked to receiving personalized PA recommendations.
2. Development of PROCare4Life digital system, including feasibility testing of the digital technologies.
3. Development and content evaluation of the PA recommendations, both by experts, researchers, and representatives of the target future users.
4. Iterative cycles of adaption of the different modules of PROCare4Life. Satisfaction evaluation of the PA recommendations for users [29,30].

The PA recommendations were included in the iterative testing from the start of pilot 2, when the technology was mature enough for testing. They were embedded in the mobile app to support users in creating and maintaining an active lifestyle. The focus of this article is to describe PROCare4Life cocreation and iterative testing processes from the PA recommendations [31,32].

### Research Aim

This article focuses on the cocreation process to support the development and iterative testing of the PROCare4Life PA recommendations and its key results. Based on the specific needs of older people living with PD or dementia, and after assessing iteratively the users’ recommendations regarding the PA contents, changes were incorporated into the digital system to improve the personalization of its end users. Overall user satisfaction assessment of the PA recommendations was also included as one of the research aims. The overall aim of PROCare4Life was to increase the QoL of people living with DD or PD, their self-management, and empowerment.

## Methods

In this section, the development of the PA recommendations, the functionalities of the PA recommendation system, and methods for pilot 2 and pilot 3 are described.

### Development

**Figure 1.**
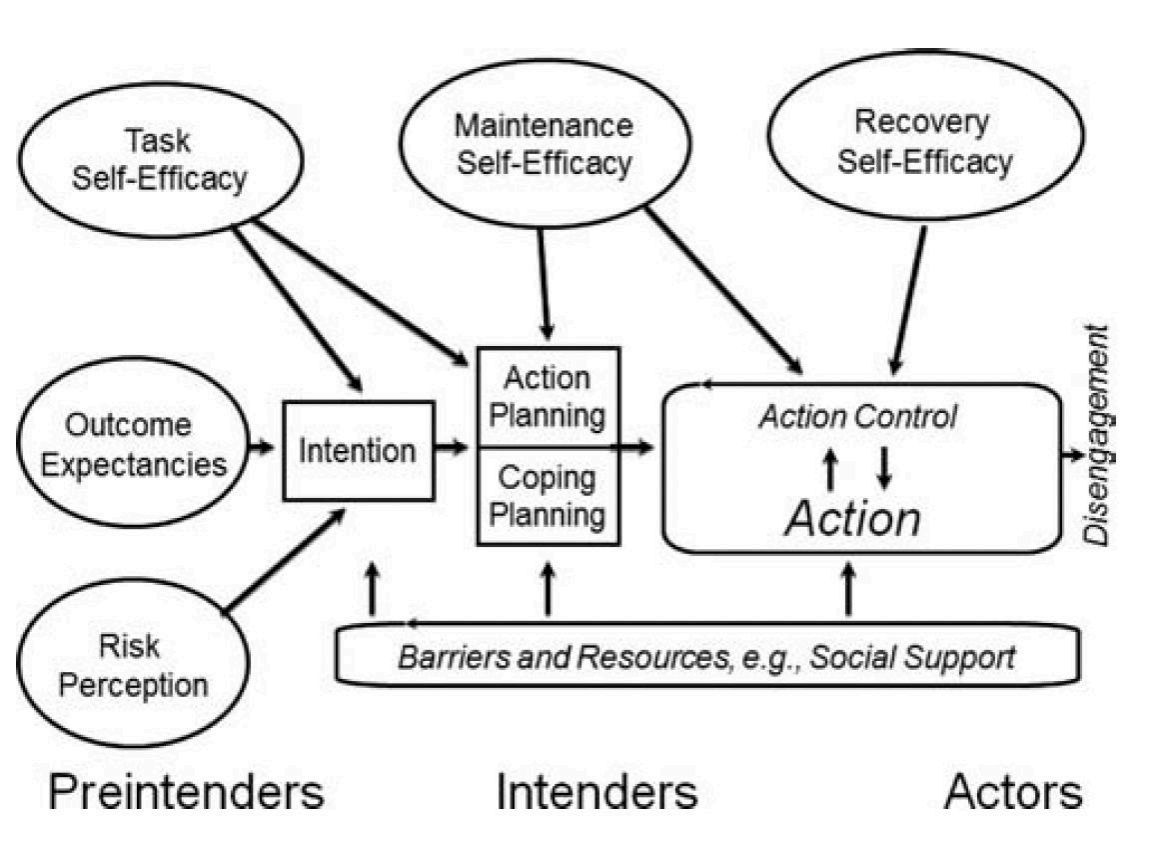
Visualization of the Health Action Process Approach.

The HAPA model was used to develop the PA recommendations of the PROCare4Life digital interactive system. It includes an initial motivational phase for goal setting and a subsequent volitional phase focusing on how to implement behavioral changes to pursue personalized goals. Between these 2 phases, individuals using the PROCare4Life system can create an intention to be active [31]. As older people living with PD or dementia, PROCare4Life users needed to have basic knowledge about the PA recommendations, including the adequate volume and intensity levels of activity for each person. Accordingly, the HAPA model was amended to include the construct Awareness of Standards, which was not originally present (Figure 1).

Four HAPA constructs of the motivational phase and 2 constructs of the volitional phase were aligned with 6 performance objectives resulting in the PA recommendation contents with 3 underlying activation factors, as summarized in Table 1 below. The contents were fused into 6 PA recommendation sets. The process to develop the PA recommendations contents is displayed in the table below (Textbox 1).

The goals of the PROCare4Life PA were reinforced by the technical architecture of the digital system, which is described in the following subsection.

**Table 1.** Development of the physical activity (PA) recommendation contents.

HAPA^a^ constructs (Activation factor)	Performance objectives	PA recommendation contents	Final PA recommendation sets
Action Self-Efficacy (Skills)	Awareness of ways to be active	Practical tips to be active	Set 1: Benefits of regular and consequences of insufficient physical activity
Outcome Expectancies (Knowledge)	Awareness of benefits of regular physical activity	Benefits of regular physical activity	Set 2: Five golden rules of physical activity
Risk Perception (Knowledge)	Awareness of consequences of insufficient physical activity	Consequences of insufficient physical activity	Set 3: Perceived exertion scale
Awareness of Standards (Knowledge)	Awareness of the physical activity guidelines	Five golden rules of physical activity, Rate of Perceived Exertion Scale, Physical activity guidelines from the World Health Organization	Set 4: Physical activity guidelines of the WHO^b^
Coping Self-Efficacy (Skills)	Experience opportunities to be active	Links for more information and videos about physical activity	Set 5: Practical tips for physical activity
Action Control (Motivation)	To be aware of your own physical activity level	Monitoring of steps via the Fitbit wristband	Set 6: Links for more information and videos about physical activity

a HAPA: health action process approach.

b WHO: World Health Organization.

Textbox 1. **Examples for the different messages of each physical activity (PA) recommendation sets.**

Set 1: Benefits of regular and consequences of insufficient PA activity

- Regular physical activity improves your sleeping patterns.
- Insufficient PA increases your risk for joint and back pain.

Set 2: Five golden rules of physical activity

- Reduce your sitting time with regular standing breaks every 30 minutes.
- Every minute of PA counts.

Set 3: Perceived exertion scale

- The Rate of Perceived Exertion Scale is a measure of subjective perceived exertion during PA with different ranges.
- Varies from very light activity (1), light activity (2-3), moderate activity (4-5) to vigorous activity (5-6).

Set 4: Physical activity guidelines of the World Health Organization

- Perform 3 activity-forms per week: Aerobic, muscle strength, and balance training.
- Conduct aerobic exercises, for example walking, 150‐300 minutes per week.

Set 5: Practical tips for physical activity

- Walking in the fresh air can be a mood booster, it improves your mind and body.
- Conducting yard work, like pulling out weed, will make your arm muscles stronger.
- Practicing this regularly, carrying groceries can be easier.

Set 6: Links for more information and videos about physical activity

- Try this link out for further information on PA: Exercises for people with dementia disease.
- Try this link out for further information on PA: Exercises for people with Parkinson disease.

### Technical Architecture

In terms of information collection, processing, and delivery, the PROCare4Life PA technical architecture consists of 2 types of components that transfer the PA recommendations as notifications to users: the low-level subsystem and the high-level subsystem.

**Figure 2.**
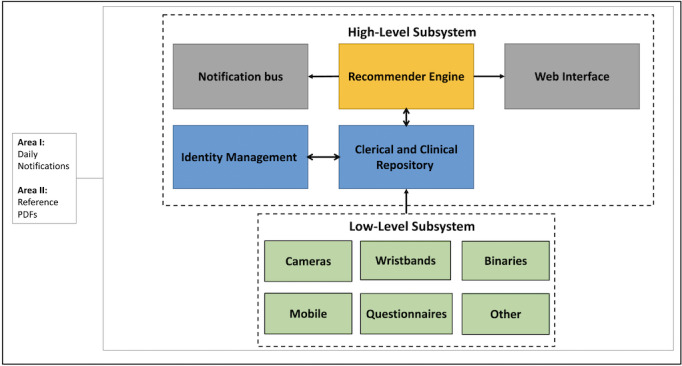
PROCare4Life (Personalized Integrated Care Promoting Quality of Life for Older People) physical activity (PA) technical architecture.

The low-level subsystem encompasses the set of devices, sensors, and questionnaires, both active and passive, functions to acquire data from patients. It requires a smartwatch, a smartphone with the included mobile health app, and questionnaires. Collected data are sent to the other subsystem. The high-level subsystem, includes the recommender engine that extracts the necessary information to generate recommendations according to each user’s profile. A notification bus sends and receives notifications involving the exchange of information and messages between the user-facing part and the systems logic based on the RabbitMQ [32] message broker. The recommender system operates on an automated daily routine, sending predefined notifications to patients, independent of their previous data. While this heuristic approach may diverge from traditional recommendation methods, it ensures consistent daily monitoring [33]. At the end of each cycle, a knowledge-based approach was used to deliver customized routines and exercises tailored to each patient’s primary disease group. Identity management provides a framework for creating, modifying, and deleting users’ data. In the Web interface, health care professionals can check historical notifications from patients and examine different patients’ information. The purpose of the clerical and clinical data repository is to store the different modalities of data that are related to users, including clinical and personal information (encrypted to comply with General Data Protection Regulation as well as some preprocessed sensorial summarized data that feeds the different dashboards and reports presented in the PROCare4Life user interface (Figure 2).

### Ethical Considerations

All pilot phases were ethically approved by the local ethical commissions of the 6 pilot sites of PROCare4Life:

- Wohlfahrtswerk für Baden-Württemberg: Approved by the Ethical commission of the University of Münster (2021-15-MB-FA2).
- Asociación Parkinson Madrid: Approved by the Ethical commission of Hospital Clínico San Carlos (21/220-E).
- Casa di Cura Policlinico: Approved by Comitato Etico Milano Area 2 of the Fondazione IRCCS Ca’ Granda Ospedale Maggiore Policlinico (OSMAMI-26/07/2021-0032326-U).
- Campus Neurologico Senior: Approved by the Comissao de Ética Campus Neurologico Senior (Ref number 3-2021).
- Spitalul Universitar de Urgenta Bucuresti and University of Medicine and Pharmacy: Approved by the Comisia de Etica e Cercatarii of the Spitalul Clinic Colentina (Number 24/28.09.2021).

As the research included human participants, the end user pilot sites prepared, within the consortium, a first draft version of the ethical documentation. All relevant information on the ethics of the study was collected. Based on the local regulations of each end user pilot site, the gathered information was integrated into the local documents, including ethical documentation for the ethics commission including the following sections: description and procedures of the study, voluntariness, and anonymity, information about no compensation type for the participation, insurance coverage, scope of data collection and processing, legal basis, option for the withdrawal, contact data of the responsible contact persons. This information was fused into the ethics application and the connected general participant information and consent. In addition, a data handling agreement was established and signed by all parties.

### Participants

PROCare4Life second and third pilots were performed in accordance with the following inclusion and exclusion criteria.

The inclusion criteria included individuals who had a clinical diagnosis of DD or PD; were 65 years or older; demonstrated a willingness to participate; had the ability to provide informed consent; and were currently living at home, in a rehabilitation center, or in a daycare center.

By contrast, candidates were excluded from participating in the study in cases of inability to learn and limitations impeding the ability to use the PROCare4Life system or key elements of its technical equipment, such as speech, vision, language, hearing, or psychiatric impairments.

Participants were recruited from the social and health care centers involved in the study (Casa di Cura Policlinico, Asociación Parkinson Madrid, Campus Neurologico Senior, University of Medicine and Pharmacy, Spitalul Universitar de Urgenta Bucuresti, and Wohlfahrtswerk für Baden-Württemberg).

### Study Procedure

The study was conducted in 3 different scenarios: neurorehabilitation center, daycare center, and at participants’ homes. People living with DD or PD received periodically personalized PA recommendations (Multimedia Appendix 1) over the 40 days that their participation lasted, via their PROCare4Life mobile app. Each day a different notification was sent. All these materials were additionally stored as PDFs (Multimedia Appendix 2) in the app allowing participants to consult them later. Table 2 summarises the PA recommendation sets by the days of their appearance, gradually covering all 6 sets.

**Table 2.** PROCare4Life^a^ physical activity (PA) recommendation sets and their appearance in the mobile health app.

Sets	Topic	Days
1	Benefits of regular and consequences of insufficient PA	1‐10
2	Five golden rules of PA	11‐15
3	Perceived exertion scale	16‐25
4	PA guidelines of the WHO^b^	26‐30
5	Practical tips for PA	31‐35
6	Links for more information and videos about PA	36‐40

a PROCare4Life: Personalized Integrated Care Promoting Quality of Life for Older People.

b WHO: World Health Organization.

#### Pilot 2

The PA recommendations were evaluated by users through intermediate evaluation questions. They appeared in the PROCare4Life mobile app itself once each set of recommendations had been completed. Additionally, the system integrated reminders that prompted patients to respond to some questions linked to each set of notifications. Users were requested to rate the information included in each PA set using a 5-point Likert scale. They were requested to select one of 5 intuitive emojis, referring to “excellent,” “good,” “average” rated as positive, “poor,” and “very poor” rated as negative.

#### Pilot 3

Over the pilot 3 implementation, participants were also invited to assess the PA recommendation system and contents after the 40-day participation, also via the PROCare4Life app-integrated mobile health satisfaction questionnaire [34]. On 12 items, they were able to select the options “agree,” “neutral,” or “disagree” (Table 3).

**Table 3.** Mobile health satisfaction questionnaire displayed in the PROCare4Life^a^ app.

Question and response number	Response
(a) What did you think about using the recommendation system in our PROCare4Life mobile app?	
Q1	It was easy to use
Q2	It was good to use
Q3	The time spent using it was acceptable
Q4	The explanation on how to use it was sufficient
Q5	It was too time-consuming
Q6	It was boring to use
Q7	It was a disturbance
Q8	I can recommend it to others
(b) How did you experience the recommendation system in your PROCare4Life mobile app?	
Q9	It has motivated me to change my lifestyle habits
Q10	It has helped me to understand the benefits of improving my lifestyle habits
Q11	It has helped me to understand how I need to change my lifestyle habits
Q12	It has helped me set personal goals for my lifestyle habits in a way that I could not have done on my own

a PROCare4Life: Personalized Integrated Care Promoting Quality of Life for Older People.

#### Statistical Analysis

The following sociodemographic data were collected for pilot 2 and pilot 3 to support the data analysis while guaranteeing the pseudoanonymization of the data: patient ID; scenarios, birth date; gender; diagnosis, and pilot site. The analysis was performed using Microsoft Excel (version 2.0). Descriptive statistical analysis was performed, identifying frequencies and percentages of responses. The evaluation results were crossed with the sociodemographic variables for comparison across diagnosis, age, pilot site, gender, and scenarios.

## Results

### Pilot 2: Content Evaluation

#### Participants’ Characteristics

A total of 167 participants were recruited, of which 55 participants were in the home scenario, 94 participants were in the rehabilitation center, and 18 participants were in the daycare center. Out of the total number of participants, 43 (26%) participants evaluated the content of the PA recommendations. Most of them (27/43, 63%) were men. The mean age was 75 (SD 5.9) years. Patients came from Spain (29/43, 67%), Italy (7/43, 16%), and Portugal (7/43, 16%). Regarding living situations, 79% (34/43) were in a rehabilitation center and 19% (7/43) were at home. Continued problems with the recruitment of patients for the daycare centers prevented the collection of significant enough information in that scenario. The most common diagnosis was PD (37/43, 86%).

#### Results Related to the Content Evaluation

The results from the intermediate evaluation questions are presented in Table 6. The first PA recommendation set evaluations had a 100% completion rate by participants. As time went by and additional sets were delivered, the number of missing evaluations increased from zero to a maximum of 10 people. Therefore, the total number of evaluations, representing 100%, differs from set to set. The first sets were mostly rated as excellent or good: “Benefits and consequences of PA” (34/43, 79.1%), “five golden rules of PA” (34/41, 83%), and “perceived exertion scale” (22/41, 54%). The same appeared by around one-quarter of users for “PA guidelines of the WHO” (9/36, 25%), “practical tips for PA” (10/35, 29%), and “content of links” (14/33, 42%). All in all, it can be clearly stated that all sets were rated by most users as positive (minimum: average). Very few people (n=5) selected the option “poor” or “very poor,” in total among all (Table 4).

**Table 4.** Results of the intermediate evaluation questions (IEQ).

	IEQ1 PAR^a^ set 1: Benefits and consequences of PA^b^	IEQ2 PAR set 2: Five golden rules of PA, n (%)	IEQ3 PAR set 3: Perceived exertion scale, n (%)	IEQ4 PAR set 4: PA guidelines from the WHO, n (%)	IEQ5 PAR set 5: Practical tips for PA, n (%)	IEQ6 PAR set 6: Content of the links, n (%)
Excellent	16 (37)	16 (39)	4 (10)	0 (0)	3 (9)	2 (6)
Good	18 (42)	18 (44)	18 (44)	9 (25)	7 (20)	12 (36)
Average	7 (16)	7 (17)	18 (44)	27 (75)	25 (71)	17 (52)
Poor	0 (0)	0 (0)	1 (2)	0 (0)	0 (0)	2 (6)
Very poor	2 (5)	0 (0)	0 (0)	0 (0)	0 (0)	0 (0)
Not reported	N/A^c^ (0)	N/A (2)	N/A (2)	N/A (7)	N/A (8)	N/A (10)
Total	43 (100)	41 (100)	41 (100	36 (100)	35 (100)	33 (100)

a PAR: physical activity recommendation.

b PA: physical activity.

c The data are marked as “not available” because it is unclear whether participants responded to the questions. Due to technical errors, we were unable to determine which responses were provided. As more sets were delivered, the number of missing evaluations increased—from zero initially to a maximum of 10 participants. Consequently, the total number of evaluations, representing 100%, varies across different sets.

### Pilot 3: Satisfaction Evaluation

#### Participants’ Characteristics

A total of 273 patients were recruited of which 132 patients were in the home setting, 102 patients were in the rehabilitation center, and 39 patients were in the daycare center. After excluding dropouts (n=36), 237 of 273 (87%) patients provided answers to the mobile satisfaction assessment questionnaire. Patients had a mean age of 72 years. Most patients came from Italy (82/237, 35%), followed by Spain (64/237, 27%), Romania (56/237, 24%), Germany (22/237, 9%), and Portugal (13/237, 5%). Regarding living situations, 120 (52%) people were located at home, 90 (38%) people were in the rehabilitation center, 22 (9%) people were in the daycare center, and 5 (2%) people did not report.

#### Results Related to the Satisfaction Evaluation

**Figure 3.**
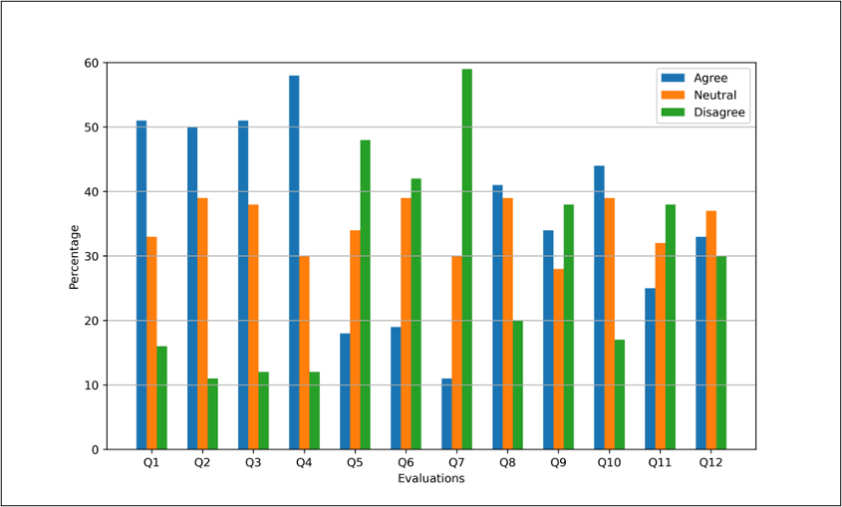
Mobile health satisfaction questionnaire results of 237 participants. Q: question*.*

Results were positive in terms of satisfaction for many of the questions linked to the PROCare4Life PA recommendations, although some were not so positive. At least half of the 237 participants agreed to the sentences “Q1-it was easy to use” (121/237, 51%), “Q2-it was good to use” (119/237, 50%), “Q3-time spent using it was acceptable” (123/237, 52%), and “Q4-explanation on how to use it was sufficient” (135/237, 57%). Compared with agreement and neutral evaluation, most people disagreed with negatively phrased worded statements, thus meaning a positive evaluation, namely “Q5-it was too time-consuming” (111/237, 47%), “Q6-it was boring to use” (99/237, 42%), and “Q7-it was a disturbance” (137/237, 58%). Most rated the following quotes positive: “Q8-I can recommend it to others” (97/237, 41%) and “Q10 it has helped me to understand the benefits of improving my lifestyle habits” (104/237, 44%). For the statement “Q12-it has helped me set personal goals for my lifestyle habits in a way that I could not have done on my own,” more people agreed (78/237, 33%) as disagreed (71/237, 30%). Most disagreement was expressed for “Q9-it has motivated me to change my lifestyle habits” (88/237, 37%) and “Q11-it has helped me to understand how I need to change my lifestyle habits” (71/237, 30%; Figure 3).

## Discussion

### Principal Findings

#### Overview

This report aimed to describe the development process of the PA recommendations according to the HAPA and activation factors, the content of the PA recommendations, and users’ satisfaction with the PA recommendation system.

All in all, the results of section 1 (Q1-Q8) of the mobile health satisfaction questionnaire clearly demonstrate that PROCare4Life PA recommendations were found to be easily understandable, interesting, and usable, and users clearly understood the benefits of PA (Q10).

Developing the contents of the HAPA model and approaching all the activation factors in information and communication technology–based interventions supported the intervention development, as stated in previous research [18,35].

#### Knowledge

The 7 statements within set 1 focusing on the positive influence of regular PA and on the consequences of insufficient PA were delivered to patients’ mobile phones. Often, older adults connect exercising directly with potential injuries and express their fear of falling [36]. After reading the first notifications, this incorrect statement can be resolved. Brawley and Latimer [37] stated that pragmatic explanations and supporting messages are necessary to facilitate regular PA. The 5 golden rules helped users to be regularly active, as adherence to the use of the app reflected. The rating of the perceived exertion scale of set 3 introduced users to the possibility of measuring their subjective perceived exertion during PA and to categorize the intensity as very light, light, moderate, or vigorous. Introducing users to the WHO’s PA guidelines within set 4, aimed to make users aware of the PA standards. Each form of training was also matched with examples of activities to increase their knowledge.

#### Skills

Statements of set 5 introduced users to practical tips for being active without specific PA equipment. Further, statements indicating the positive influences of PA on one’s daily life within set 1 were sent to the users to perform the PA activities. The final 5 notifications of set 6 gave users access to links for more information and videos about PA based on the diagnosis and further explanations in the local language.

#### Motivation

The Fitbit wristband helps users in monitoring their number of steps to keep their motivation on a high level.

The PROCare4Life system chose to incorporate a mobile health app and Fitbit because PA recommendations may be better followed and lead to less sedentary lifestyles when they incorporate technology, due to the possibility to personalize the health values [38].

Interpreting the results of Q9-Q12 of the mobile health satisfaction questionnaire shows that the recommendation system is critical on how to motivate to change lifestyle habits, to set personal goals, and to facilitate these. The interpretation of these results is not completely clear to the research team. One possible conclusion was that the app failed to achieve its goal of improving the PA among participants and in changing their lifestyle habits. Another alternative explanation is that the participants were already motivated and understood how to change their lifestyle habits, and thus the reasons behind the negative assessment of these 2 questions might reflect an already positive starting point for the individuals sharing their disagreement. Further research might be needed in this respect. Features that could counter the criticism are more personalized features that address the remaining constructs of the HAPA that are currently missing: By addressing the construct Action Planning of the HAPA, users could have the option to select a personal goal of steps based upon their measured average number of weekly steps, differentiated into maintenance (average base), low ambition (average base +10%), medium ambition (average base +20%), and high ambition (average base +30%). Lee et al [39] roofed that technology-based goal setting helps people to advance the effectiveness of the number of steps.

By focusing on the construct Action Coping of the HAPA, the system could recommend specific “exergames” via smart TV to the user, focusing on their measured average number of weekly steps, and on days with unsuitable weather for outdoor activity. The findings of Rhodes et al [40] highlight the social aspects of exergaming. Exergames are capable of incorporating more than one user, to keep the motivation on a high level. Addressing the construct Recovery Self-Efficacy of the HAPA, the system could send users an underachievement notification (eg, “This week you did not achieve your personal step goal. You took 900 steps on average. Don’t worry, you can try it again or change your personal goal for steps”) [41]. Future interventions are encouraged to consider addressing the remains of the HAPA construct, facilitating more personalized PA interventions.

### Limitations

The period in which pilot 2 of PROCare4Life was carried out was challenging due to COVID-19 and analysis of its outcomes highlighted some limitations of the system. Specifically, pilot 2 experienced installation and setup issues, including smartphone and the Fitbit. Difficulties with the initial setup prevented the patients from using the system at all, which also implied that PA recommendations were not used nor evaluated. Consequently, one-quarter of the participants were represented in the pilot 2 data.

To counteract changes performed in the solution deployment to reduce the complexity of the setup and improve the user experience. The main change was the elimination of the physical mini-PC, which was the data collection hub for pilot 1 and pilot 2, and at the same time, the main source of installation and operation issues. In pilot 3, most of the patients only had the wristband and the phone. Data collection figures increased significantly in pilot 3. Additional changes were made to the PA recommendation components of the PROCare4Life solution, enabling more data collection points and potentially a more interactive experience for the patients.

This study reported on the theoretical background of behavior change techniques and on their technical development. The results are based on multinational participants, across various settings and health conditions. The multidisciplinary team, which included technical partners (for developing the backend and front end), academic partners (for creating research-related content), an educational network (for producing relevant materials), a security company (for guaranteeing data protection and privacy requirements) as well as clinical pilot sites (for testing the end product), followed an iterative design process for developing the interfaces as recommended by Zaman et al [42].

### Conclusions

The PA recommendations were implemented in a systematic way and addressed the activation factors like skills, knowledge, and motivation to focus on relevant behavior change constructs. Based on users’ perceptions of the system, the content was rated positively, and users were satisfied with the PA recommendations. Addressing the activation factors can be recommended for researchers and technical developers of other projects. The current implementation of the mobile health app uses daily static notifications and performs limited monitoring of patients’ activities. More features need to be implemented to personalize the recommender system to each individual user.

## Supplementary material

10.2196/51831 Multimedia Appendix 1 Physical Activity Recommendations daily messages.

10.2196/51831 Multimedia Appendix 2 Physical Activity Recommendations in PDF files.

## Abbreviations

DD: dementia disease
EU: European Union
HAPA: Health Action Process Approach
PA: physical activity
PD: Parkinson disease
PROCare4Life: Personalized Integrated Care Promoting Quality of Life for Older People
QoL: quality of life
