# Supplementary material for: Cocreating a Mobile Health App Providing Physical Activity Recommendations for Older People Living With Parkinson Disease or Dementia: User-Centered Pilot Study
Source: JMIR Form Res. 2025 Jun 19;9:e51831. doi: 10.2196/51831 (PMC12202238; doi:10.2196/51831)
Supplement: Multimedia Appendix 1 [file formative-v9-e51831-s001.docx]

This is a Multimedia Appendix to a full manuscript published in the J Med Internet Res. For full copyright and citation information see <http://dx.doi.org/10.2196/jmir.xxxx>

Multimedia Appendix 1: Physical Activity Recommendations daily messages

Benefits of regular and consequences of insufficient physical activity:

1. Regular physical activity improves your physical abilities.
2. Regular physical activity reduces your risk of fall.
3. Insufficient physical activity can be a reason for depressive mood and loneliness.
4. Regular physical activity helps you to manage stress.
5. Regular physical activity improves your sleeping patterns.
6. Insufficient physical activity increases your risk for joint and back pain.
7. Regular physical activity improves your mobility and balance.
8. Regular physical activity improves your heart and respiratory performance.
9. Insufficient physical activity is one of the indirect leading factors for death worldwide.
10. Regular physical activity improves your quality of life.

Five golden rules of physical activity:

1. Try to be as physically active as your abilities and conditions allow.
2. Try to perform activities in short bouts of 10 minutes duration.
3. Reduce your sitting time with regular standing up breaks every 30 minutes.
4. Include physical activity in your daily life activities based on the recommended values.
5. Know that every minute of activity counts.

The Rate of Perceived Exertion Scale:

1. Is a measure of subjective perceived exertion during physical activity with different ranges.
2. Varies from very light activity (1), light activity (2-3), moderate activity (4-5) to vigorous activity (5-6).
3. Defines “very light activity (1)” as any activity other than complete rest.
4. Defines “light activity (2-3)” as it feels like you can maintain it for hours while easily breathing.
5. Defines “moderate activity (4-5)” as you can hold short conversations while exercising.
6. Defines “vigorous activity (6-7)” as feels like you on the verge of uncomfortable with short of breath.
7. Includes as “very light activities” for example: Folding laundry or washing dishes.
8. Includes as “light activities” for example: Walking slowly or vacuuming.
9. Includes as “moderate activities” for example: Walking briskly or swimming.
10. Includes as “vigorous activities” for example: Jogging or carrying heavy loads.

Physical Activity Guidelines from the World Health Organisation:

1. Perform three activity-forms per week: Aerobic, muscle strength and balance training.
2. Conduct aerobic exercises, for example walking, 150-300 minutes per week.
3. Conduct muscle strength exercises, for example lifting weights, on 2 days per week.
4. Conduct balance exercises, for example heel raises, on 3 days per week.
5. Combine aerobic, muscle strength and balance exercises in your physical activity weekly routine.

Practical tips for physical activity:

1. Walking in the fresh air can be a mood booster, it improves your mind and body.
2. Household activities, like vacuum cleaning or wiping keep you fit and independent.
3. Conducting yard work, like pulling out weed, will make your arm muscles stronger. Practicing this regularly, carrying groceries can be easier.
4. Performing heel raises while holding on to a chair back can help you to improve your balance. Practicing this regularly, it can prevent falls.
5. Going up and down the stairs, by holding on the rail, will make your leg muscles stronger. Practicing regularly, sitting down and standing up will be easier for you.

Links for further information to physical activity:

1. Try this link out for further information to physical activity.
2. Try this link out for further information to physical activity.
3. Try this link out for further information to physical activity.
4. Try this link out for further information to physical activity.
5. Try this link out for further information to physical activity.
