# Supplementary material for: Cocreating a Mobile Health App Providing Physical Activity Recommendations for Older People Living With Parkinson Disease or Dementia: User-Centered Pilot Study
Source: JMIR Form Res. 2025 Jun 19;9:e51831. doi: 10.2196/51831 (PMC12202238; doi:10.2196/51831)
Supplement: Multimedia Appendix 2 [file formative-v9-e51831-s002.docx]

This is a Multimedia Appendix to a full manuscript published in the J Med Internet Res. For full copyright and citation information see <http://dx.doi.org/10.2196/jmir.xxxx>

Multimedia Appendix 2: Physical Activity Recommendations in pdf files

1.
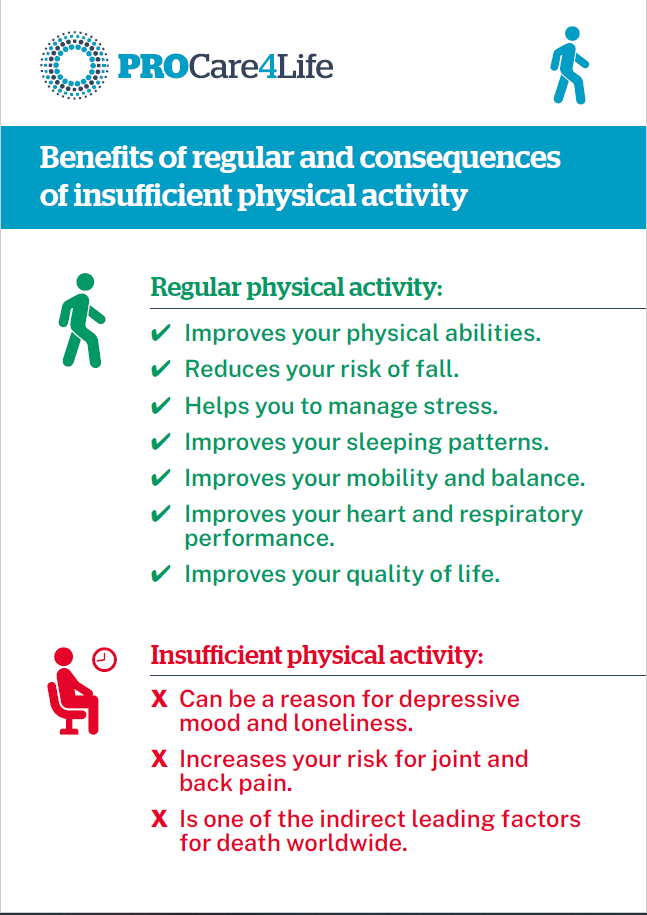
Benefits of regular and consequences of insufficient physical activity
2. **
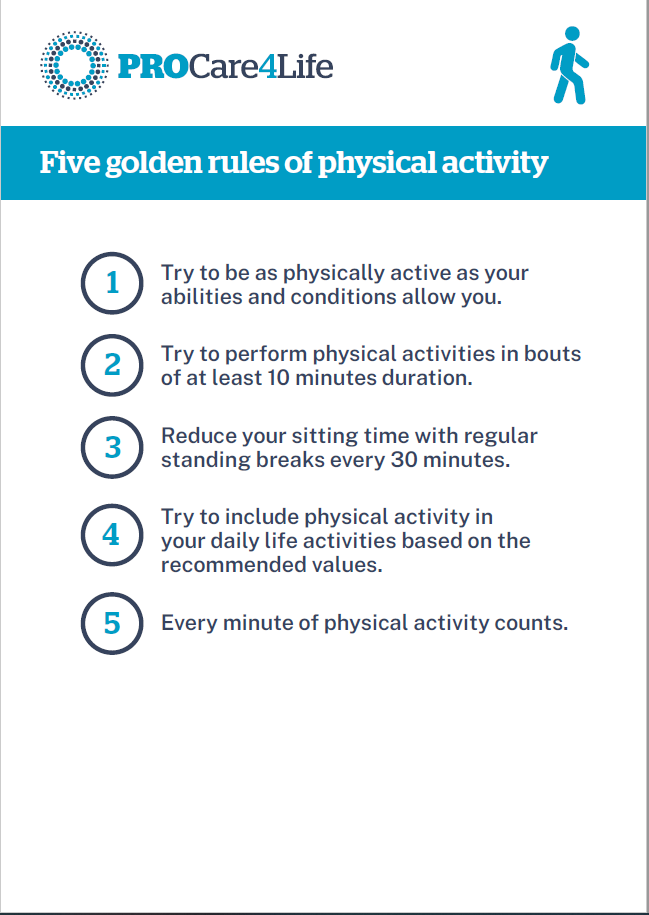
**Five golden rules of physical activity
3.
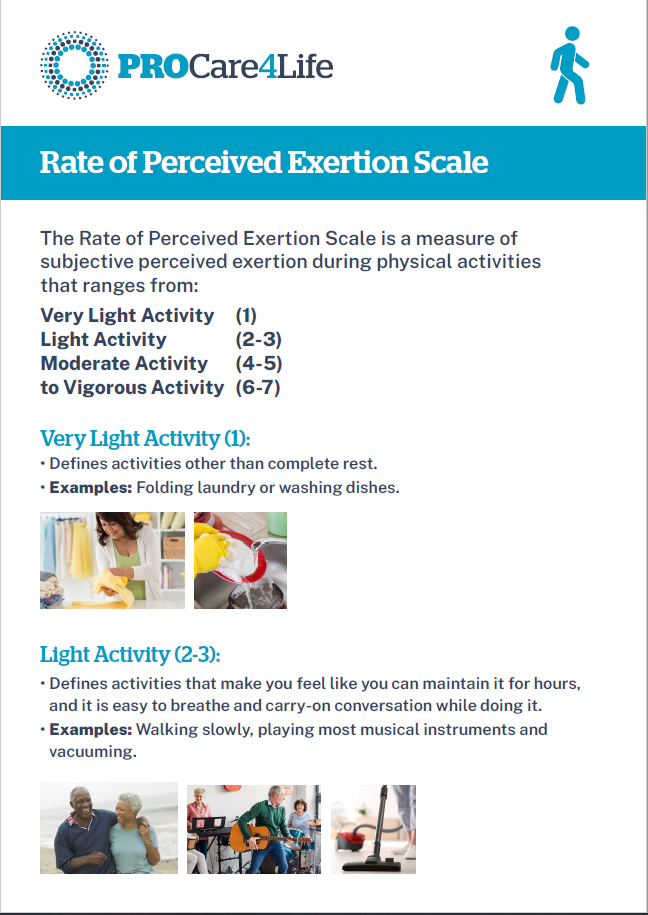
Rate of Perceived Exertion Scale

**
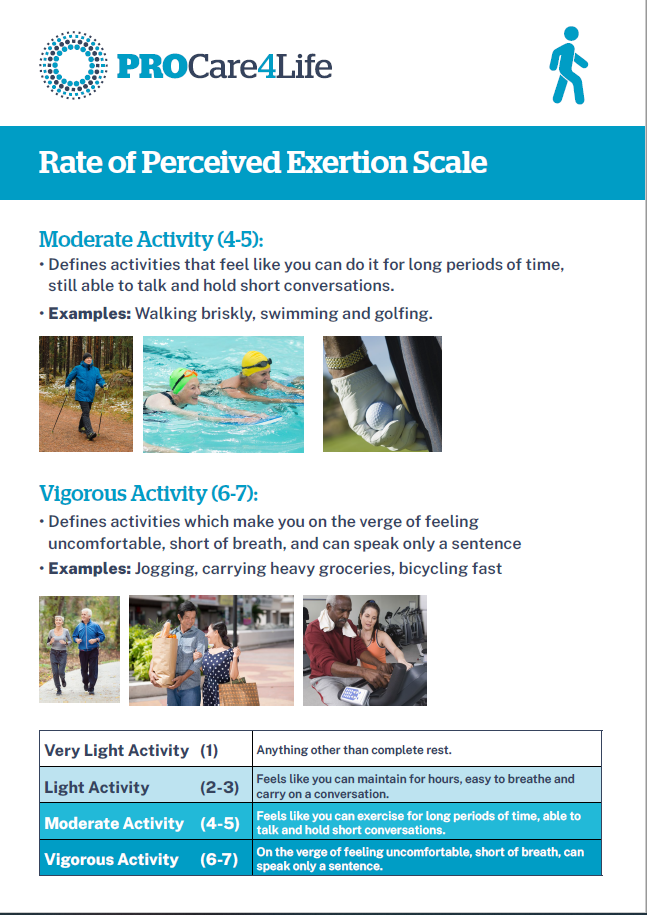
**

1.
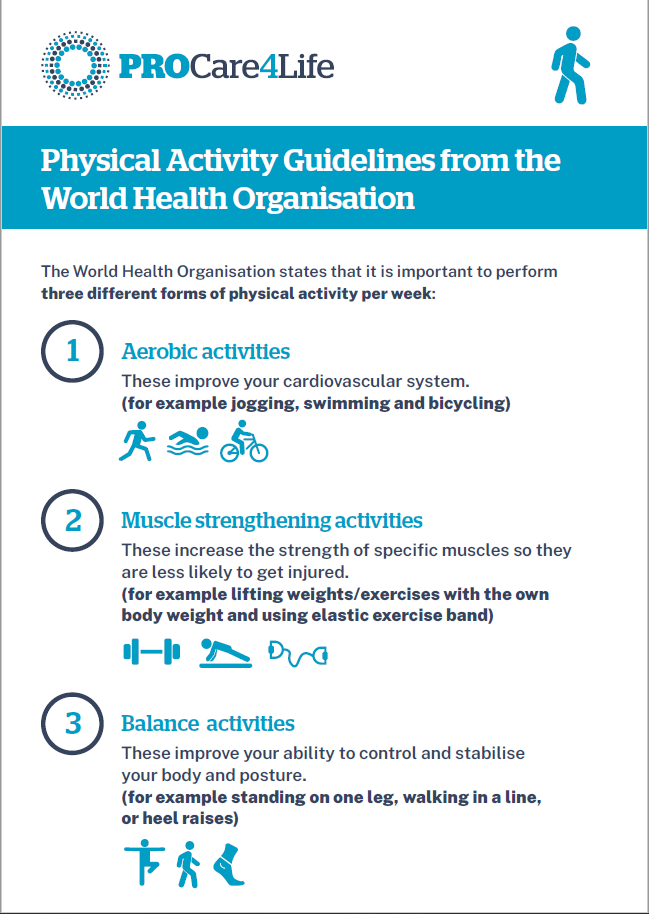
Physical Activity Guidelines from the World Health Organisation

**
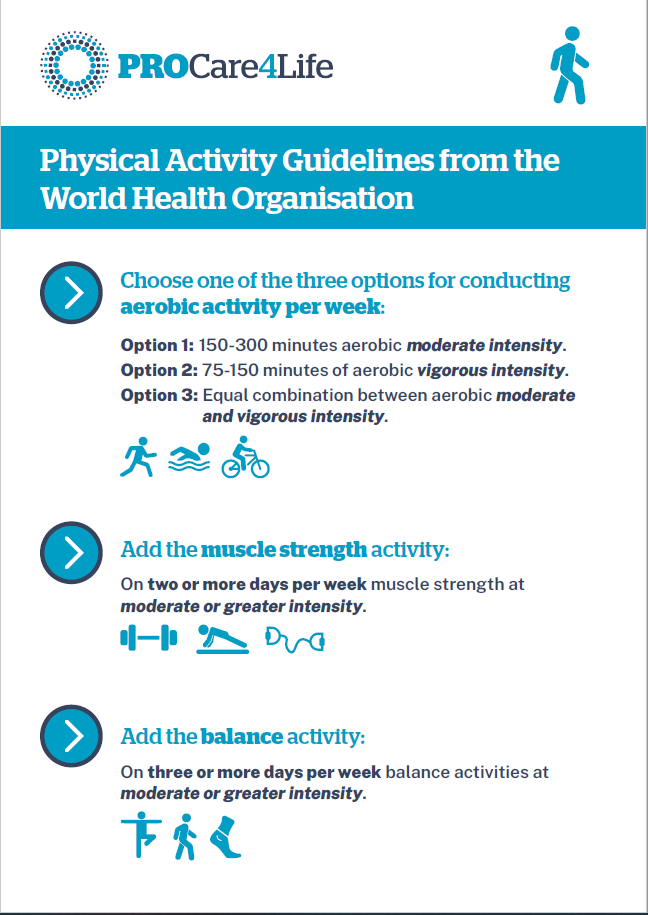
**

1. **
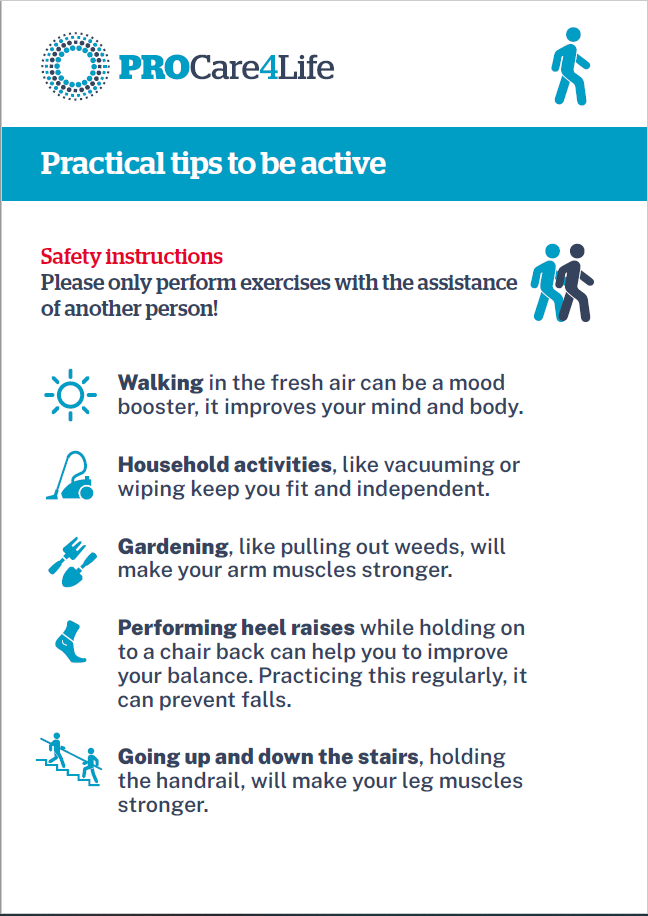
**Practical tips to be active:
2. **
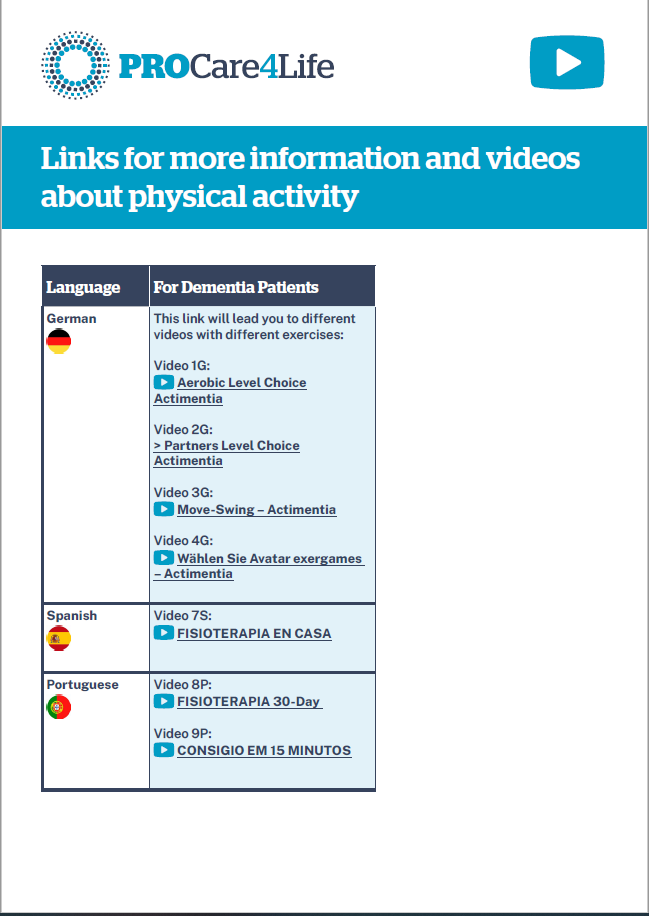
**Links for more information and videos about physical activity
